# Amick’s “Press Dispatches”

**Published:** 1893-11

**Authors:** 


OUR Galveston collaborators must be excused this month, as they were all heavily taxed for the Pan-American Medical Congress, and as the labor incidental to the opening of the regular session of the Medical College took much of their time, they were not able, with the exception of Prof. Thompson, to find time to prepare matter for their respective departments. In our next issue, the Christmas edition, we hope to make ample amends.

